# Supplementary material for: Financial frictions and stock return: A novel least minus more frictional factor for asset pricing models in emerging economies
Source: PLoS One. 2025 Jul 14;20(7):e0325917. doi: 10.1371/journal.pone.0325917 (PMC12258585; doi:10.1371/journal.pone.0325917)
Supplement: Attachment — Submitted filename: rewiew.docx [file pone.0325917.s002.docx]

The reviewer believes that this paper has made a significant innovative marginal contribution to the literature by introducing financial friction factors to explain stock returns based on four types of capital asset pricing models (CAMP).

My concerns, comments and suggestions are as follows:

Questions with the definition of variables in Table 1

1 There is no definition of "N" in SMB, and what does S/L refer to?

2 What does Winner Minus Loser (WML) mean, and what does S/W mean?

3. The subscript 1-5 in LMM is ambiguous, does it refer to five types of friction, but the paper only mentions four types of friction generated at the macro, micro, financial market, and corporate level. Is it a combined friction? How to synthesize?
4. How are Small-Big, High-Low, and Conservative-Aggressive defined?

The above variable measures are an important basis for subsequent panel regression.

5. Panel data regression involves the selection of mixed, random effects, and fixed-effect models, and can also consider the heteroskedasticity of disturbed terms, autocorrelation test, and cross-section correlation test. If you use STATA, you can run the following command:

Cross-section correlation tests: xtcsd, fre

Heteroskedasticity test: xttest3

Autocorrelation test: xtserial

If the above problems exist, the panel data regression can be corrected by xtscc.
