# Supplementary material for: Financial frictions and stock return: A novel least minus more frictional factor for asset pricing models in emerging economies
Source: PLoS One. 2025 Jul 14;20(7):e0325917. doi: 10.1371/journal.pone.0325917 (PMC12258585; doi:10.1371/journal.pone.0325917)
Supplement: Attachment — Submitted filename: Response to Reviewers.docx [file pone.0325917.s004.docx]

**Reviewers' Suggestions and Corrections in the Research Paper**

**Financial frictions and stock return: A novel least minus more frictional factor for asset pricing models in emerging economies**

**Manuscript ID: PONE-D-25-10692**

The authors of the paper are indeed thankful to the editor and reviewers for their valuable comments. All the observations of the reviewer and editor are fully addressed in the revised version of the paper.

| **Reviewer Comments** | | |  |
| --- | --- | --- | --- |
| **Reviewer-1 Comments** | | **Corrections** | **Reference Line Numbers** |
| 1. | It is not clear on how the least minus more frictional factor approach is better than traditional approaches, e.g., Bali et al. (2016), to price if the friction factors are priced in stock market. In addition, there is a huge literature that firm characteristics such as size is a proxy for financial frictions, see Ferreira et al. (2023) and reference therein. It is therefore important to properly test your frictional factor with traditional approaches that are more robust than the authors' current approach. | This study aims to directly measure specific frictions (e.g., macro, micro, and financial market frictions) rather than relying on indirect proxies like size and market capitalization. This direct measurement can potentially capture nuances that broad proxies might miss. We acknowledge the recent extensive literature utilizing firm characteristics as proxies for financial frictions, including the important work of Ferreira, Haber (1) and Bali, Brown (2), however, our approach offers a potentially more granular and specific lens on the impact of particular frictions. Firm characteristics such as size can be influenced by various factors beyond financial constraints, making it challenging to isolate the precise effect of friction. Therefore, to counter this issue we run traditional Fama-McBeth regression on frictional factors to determine whether a proposed asset pricing model can explain the differences in average returns across a set of assets at a given point in time. | 293-303 |
| 2. | in regard to the macroeconomic frictions, the authors discuss that macroeconomic policies can either mitigate or amplify the consequences of frictions. On the other hand, Baker et al. (2016) have shown the impact of Economic Policy Uncertainty to economy, and their data is available to China and India. In this line of research, Kundu and Paul (2022) recently report its impact to stock market of G-7 countries. Therefore, the authors may need to consider it whenever available to see if it mitigates or amplifies the consequences of these frictions to stock market. | As highlighted by Baker et al. (2016), Economic Policy Uncertainty, EPU represents a significant macroeconomic friction that can profoundly impact economic activity. Their valuable dataset, now available for China and India, offers a compelling avenue for further investigation within our framework. Moreover, the recent work by Kundu and Paul (2022) on the impact of EPU on G-7 stock markets underscores the relevance of this factor in understanding market dynamics.  Building upon this literature, future iterations of our research will aim to incorporate measures of EPU for the specific economies under examination, where data availability permits (e.g., China and potentially India). This will allow us to directly evaluate the interplay between macroeconomic policies and the identified macroeconomic frictions. Specifically, we intend to explore whether periods of heightened EPU intensify or mitigate the consequences of these frictions on stock market behavior. | Foot Note 2 |
| 3. | any reason to only focus on the manufacture firms? the authors should consider all the firms publicly listed in each of 5 countries. if the frictions have the market-wide impact, we should observe similar effects to non-manufacture firms. | The sample countries are dominated with the manufacturing sector as compared to the services sector and have capital- and labor-intensive industries (3). The manufacturing firms were selected for analysis due to its pronounced sensitivity to financial frictions, whereas the financial sector is posited as the transmission mechanism and conduit for these constraints. | Foot Note 1 |
| 4. | it is not clear why data excludes 2019-2020 period since stock market is still open in that time period. | As Covid-19 period is characterized by unprecedented levels of volatility, significant market disruptions, and potentially irrational investor behavior directly linked to the unfolding COVID-19 pandemic (4). Including this period, with its outlier characteristics, could potentially skew our results and obscure the underlying long-term relationships we aim to investigate. By excluding these exceptional years, we aim to provide a more robust and stable analysis of the typical market dynamics. | 381-388 |
| 5. | in Equation 11, RMRF should be the expected market risk premium, not average of market returns less risk-free rate. In equation 12 to 26, since your LHS variable is expected value, the RHS shouldn't have the error term. | In Equation 11 mentioned error is corrected by replacing ${ER}_{i}$ by $R_{it}-{RF}_{it}$which is the return on a portfolio in excess of one month risk-free rate individual asst *i* at time *t.*  Since LHS has is excess market returns according to Fama and French (5) and Carhart (6) asset pricing models error term is supposed to be a part of RHS. | In all the equation this change in incorporated |
| 6. | not sure how to read Figure 2 to 4, especially the panel on the bottom right of each Figure. The authors may need to consider another way of data visualization. | A pilot study is performed on the manufacturing firm data of China, Inda and Pakistan by using the same linear models as used by Fama and French (7), Carhart (6), and Fama and French (5) asset pricing models. The results of pilot study are not mentioned due to redundancy however their graphs are presented here to depict the trend of stock return in these assets pricing models. The Fig. 2, Fig. 3, and Fig. 4 shows the expected return on the manufacturing firm of China, India and Pakistan. In this analysis CAPM, Fama and French three factor (FF3F) model, Carhart four factor model (Carhart 4F), and Fama and French five factor (FF5F) models. Comparison is presented with and without least minus more, LMM frictional factor. The illustrations show that these models highlight significant inclination and overestimation of asset pricing models without LMM factor as compared to the asset pricing models with LMM. Moreover, these models also follow the same pattern with and without LMM i.e. when LMM Factor is not incorporated these asset pricing models shows higher return. However FF5F gives little diverse outcome. The precise impact of LMM factor is measured in the main study models with fixed and random effect models. This pilot study by using traditional asset pricing models allowed us to further incorporate least minus more, LMM factor in the existing models. | 569-578 |
| **Reviewer-2 Comments** | | **Corrections** |  |
| 1 | Questions with the definition of variables in Table 1  1 There is no definition of "N" in SMB, and what does S/L refer to? | Mentioned terms are further explained in Table 1 as “S/L is the portfolio of small firms with lower value, B/L is the portfolio of big firms with lower value, S/N is the portfolio if small firms with neutral value, S/H is the portfolio of small firms with higher value. Similarly, S/W is the portfolio of small firms with winning return, S/L is portfolio of small firms with looser return, B/W is the portfolio of big firms with winners return and B/L is the portfolio of bigger firms with looser return. SC is the portfolio of small firms with conservative investment return and BC is the big firms with conservative investment firms. SA is the small firms with aggressive investment and BA is the big firms with aggressive investment strategies.” | Table 1. Note |
| 2 | 2 What does Winner Minus Loser (WML) mean, and what does S/W mean? | Mentioned is term is further elaborated as “SMB is the excess return on the portfolio of small caps firms less the big caps firm. *HML* is the excess return on higher valued firms minus the lower valued firms. *WML* is the excess return on the portfolio of firms which were winning in the last period less looser firms. *CMA* is the excess return of firms with conservative investment less the firms with aggressive investment strategies.” | It is explained in Table 1 Note |
| 3 | 3. The subscript 1-5 in LMM is ambiguous, does it refer to five types of friction, but the paper only mentions four types of friction generated at the macro, micro, financial market, and corporate level. Is it a combined friction? How to synthesize? | LMM is constructed with four types of friction i.e. macro-, micro-, financial market and firm-level frictions. However, in this study a fifth type i.e. international level friction, is also described but since import and export is not the scope of this study this type of friction is not included in this study. Yes, LMM is a combination of four types of frictions. Each friction is computed separately by using previous studies proxied and measures and their combining effect is incorporated in this study as least minus more, LMM frictional factor. | This explanation is not included in article. |
| 4 | 5. Panel data regression involves the selection of mixed, random effects, and fixed-effect models, and can also consider the heteroskedasticity of disturbed terms, autocorrelation test, and cross-section correlation test. If you use STATA, you can run the following command:  Cross-section correlation tests: xtcsd, fre  Heteroskedasticity test: xttest3  Autocorrelation test: xtserial  If the above problems exist, the panel data regression can be corrected by xtscc. | Yes we have run panel data regression by using STATA.  Pesaran test is applied on the data to test the Cross-section correlation among the study variables. However, for cross-section correlation of error terms Hausman Test is applied.  To test autocorrelation Wooldridge test is applied. Durbin Watson test is also applied on the data to check autocorrelation. Data skewness, and kurtosis are also checked to identify the outliers and data trend.  Yes, mentioned commands are also used in STATA for the said test. | 609-610  589-591 |
| **Reviewer-3 Comments** | | **Corrections** |  |
| 1 | Although the article introduces the concept of financial friction, it lacks in-depth analysis of the relationship between different levels of friction. For example, the application of agency theory only stays at the theoretical level and has not been fully validated with empirical data. | **The relationship financial frictions in the light of agency theory is further explained as follows:**  Higher levels of information asymmetry (financial market friction) a key aspect of financial friction can intensify the principal-agent problem, leading to increased monitoring costs and potentially suboptimal investment decisions (8). Conversely, reduced information asymmetry, perhaps through improved transparency mechanisms (9), might mitigate these agency costs even in the presence of other forms of financial friction, such as transaction costs. Furthermore, this study recognizes the need for empirical validation while the current draft primarily lays theoretical groundwork, we propose two avenues for addressing this in the revised manuscript. First, we will incorporate a more thorough review of existing empirical literature that examines the interplay between financial friction and agency theory. Study by Hadlock and Pierce (10) examines financial constraints and investment provide valuable insights into how financial constraints (a form of friction) affect corporate behavior in line with agency cost arguments. This study explicitly discusses how our theoretical framework aligns with and potentially extends these empirical findings.  Second, this study outlines potential avenues for future empirical research that could directly test the relationships we propose. This involves suggesting specific datasets and econometric methodologies that could be employed to quantify the impact of different levels of financial friction on agency-related outcomes, such as executive compensation structures or corporate governance mechanisms (8). | 175-202 |
| 2 | The data sample is limited to five emerging economies in South Asia, which may limit the generalizability of the research conclusions. Expanding the sample size or increasing the selection of sample data demonstrates the universality of research conclusions. | Since Pakistan is the main focus of the study and its comparison is made with the neighboring countries. Therefore, this study gives an interesting case of South Asian select economies. In this study two countries China and India are actively growing economies, and two countries are relatively stalled economies i.e. Bangladesh and Sri Lanka as compared to Pakistan. Moreover, all the economies have same ecological and geographical dynamics which make comparison easier, and if we include any other country from like US, Canada, Australia, Russia or European countries then it would be difficult to make caparison with such a diverse and advanced economies. | 147-154 |
| 3. | Although a panel data model was used, potential endogeneity issues were not addressed. | Data stationarity is checked in the initial pretesting and validation of the data process to remove endogeneity issue at the first difference. While direct testing for endogeneity is often difficult, the Hausman test which is performed in the Diagnostic Test Section 7.1 which provide indirect evidence of correlation between regressors and the unobserved individual effects which is relevant when choosing between Fixed effect and random effect models.  Based on the suspected sources of endogeneity and the characteristics of our data, static models (Estimated generalized least square) vs dynamic models (Fixed effect and Random effect) in the light of available instruments are selected. The results of the most appropriate models are then applied. For instance, in the case of China, India, and Pakitan Fixed effect was dominated so fixed effect model results are used. Similarly in case of Sri Lanka random effect model result are more significant so random model is used. While the results of Bangladesh were not significant in all the mentioned models as it has more autocorrelation and endogeneity issues (This mentioned details is already incorporated in the main text of the manuscript in the description of Table 7 results). |  |
| 4. | The results from Bangladesh are mostly not significant, but the study did not explain this difference, weakening the generalizability of the conclusions. | Since the results are presented for each country separately and their cross comparison is made. Therefore, the insignificant results of Bangladesh do not affect overall generalizability of the conclusion section. If we could have run overall and combined result of all the countries, then this could have an effect on or weaken the generalizability of the conclusion section. |  |
| 5. | The conclusion of the article is relatively vague and does not fully integrate empirical results for in-depth analysis. The policy recommendations are relatively broad, lacking specificity and operability. | The conclusion section is further enhanced with the following specific explanations regarding alpha, SMB, HML, WML and LMM factor results.  By including frictional factor the alpha value is significantly reduced in all the asset pricing models which suggest that the portfolios are generating less return than what would be expected based on their exposure to the model's factors. It indicates a decline in the manager's ability to generate returns above the benchmark predicted by the models. Alpha is often attributed to a manager's skill in selecting undervalued stocks (stock selection) or in anticipating market movements (market timing). A decreasing alpha could imply a deterioration in these skills or that the strategies employed are no longer as effective in the current market frictional conditions (11). The market factor in traditional models assumes frictionless trading. Introducing friction can reduce the explanatory power of the market factor because friction distorts arbitrage, preventing efficient pricing and making some assets appear riskier than they are under perfect markets (12). Investors demand additional compensation for bearing liquidity risk or transaction costs, which is not captured by market beta alone (13).  When frictional factors are introduced, smaller companies, which are often less liquid, could be more heavily impacted by transaction costs, which may diminish their premium or render it less predictable (14). Therefore, the SMB factor could lose significance in a model that accounts for such costs.  Similarly, when frictional factors are considered, the relative performance of value stocks might be less pronounced, as buying and selling these stocks might incur higher costs. Thus, value stocks might not always outperform as expected, especially when these costs eat into potential returns (15). Financial frictions can erode the profitability of momentum strategies by increasing trading costs as mentioned by de Roon and Szymanowska (2012), who show that incorporating transaction costs into asset pricing models reduced the predictability of returns from momentum strategies. They found that transaction costs as low as 35 basis points could reconcile the observed return predictability with asset pricing models. Same results are reported by the Endri and Endri (2020) who incorporates transaction costs into the traditional Fama-French three-factor model. Their findings indicate that while the traditional factors (market, size, and value) remained significant, the inclusion of transaction costs provided a more accurate representation of expected returns, especially for stocks with higher trading frictions.  **The following specific and objective policy recommendations are suggested according to the results and conclusion section.**  Governments should focus on reducing inflation and interest rates to minimize financial market friction. High inflation increases uncertainty, erodes the value of money, and increases transaction costs. Lower interest rates will reduce borrowing costs for firms, encouraging investment and improving overall market efficiency. Implement monetary policies aimed at controlling inflation and keeping interest rates stable. This will make financing cheaper and reduce liquidity risks, improving firms’ ability to invest and grow.  Simplify tax and tariff structures for manufacturing firms to reduce financial constraints. Financial frictions negatively impact firm performance due to government interventions like taxes and tariffs such as lower corporate taxes, streamline tariffs, and provide tax incentives for firms investing in R&D and technology upgrades. This would enable firms to better manage operational costs and enhance their competitiveness. Introduce regulations aimed at enhancing market transparency, improving financial reporting standards, and encouraging the adoption of modern technologies. This will make the market more efficient and reduce the impact of financial friction on firms' stock returns.  Emerging economies need robust financial systems to better absorb financial shocks and reduce the negative impact of liquidity risk on asset pricing for strengthening financial institutions, promote capital market development, and encouraging foreign investment. This will enhance the depth and liquidity of local markets, enabling firms to access better financing options and reducing the overall cost of capital.  Results shows that smaller firms are particularly vulnerable to transaction costs and financial frictions. Providing better access to capital will allow these firms to grow and become more efficient. Therefore, government should develop policies to improve access to external financing, especially for Small and Medium Enterprises, such as low-interest loans, venture capital funds, or government-backed guarantees for business loans. This will help them overcome capital constraints and reduce the impact of liquidity risk on their operations. | 814-841  924-947 |
| 6 | The article's discussion of research limitations is relatively simple and does not fully consider possible alternative explanations or improvement directions. | The limitations identified highlight several areas for potential improvement and deeper exploration in future research. Firstly, the study’s reliance on a limited number of metrics to examine macroeconomic and microeconomic frictions focusing mainly on external financing and financial constraints may overlook other critical dimensions of financial frictions such as information asymmetry, legal enforcement, governance quality, and transaction costs. Future studies could improve the robustness of the analysis by incorporating a broader set of variables or constructing a composite index of financial frictions that include internal financing constraints, corporate governance indicators, and market transparency measures.  Secondly, the geographical scope of the study is restricted to emerging economies in South Asia, which limits the generalizability of the findings. Since emerging markets differ widely in their economic structures, regulatory frameworks, and financial systems, expanding the analysis to include other regions such as Latin America, Eastern Europe, or Sub-Saharan Africa would provide a more comprehensive understanding of how financial frictions operate across various contexts. A cross-regional panel analysis could also uncover whether the observed effects are region-specific or universally applicable.  Another significant limitation stems from data incompleteness and inconsistency, particularly affecting firms in Bangladesh and Sri Lanka. Many companies were excluded due to recent listings or delisting’s, which may have introduced selection bias into the sample and reduced the representativeness of the findings. To address this, future research should seek alternative or complementary data sources—such as firm-level surveys, private databases, or public filings—and apply data imputation techniques or bootstrapping methods to mitigate the impact of missing data.  Lastly, the small sample size in certain countries weakens the statistical power and may lead to unreliable estimates. This issue can be alleviated by extending the time horizon of the data, utilizing more granular data (e.g., monthly or quarterly), or adopting statistical techniques suitable for small samples, such as Bayesian estimation or shrinkage methods. Additionally, applying firm-level segmentation based on industry, ownership, or age could reveal heterogeneous effects of financial frictions that a pooled analysis may obscure. Overall, by addressing these limitations through broader data collection, expanded geographic coverage, and enhanced methodological rigor, future research can produce more generalizable and insightful findings on the role of financial frictions in asset pricing and firm performance. | 909-930 |
| **Reviewer-4 Comments** | | **Corrections** |  |
| 1 | The analysis of empirical results lacks clarity, particularly in Sections 7.2.1 to 7.2.4. The authors are encouraged to present their findings in a more structured and logical manner. A clearer exposition would significantly improve the readability and interpretability of the results. | Results are further refined, revised and elaborated by following the Fama and French (7), Carhart (6), and Fama and French (5) results presentation methodology. Now alpha, RMRF, SMB, and HML results are presented as a combined in 7.2.1 section. While momentum WML, and LMM factor results are presented in 7.2.2 and 7.2.3 sections.  In China when LMM factor is introduced in the CAPM model alpha value is reduced by 19.2%, 5% it is reduced in India, 4.3% it is reduced in Pakistan, and 4.7% it is reduced in Bangladesh. Alpha values are significant in all the models and countries except for Bangladesh. In FF3F model alpha value is reduced by 4% in China, while it remains same in India and Sri Lanka and it reduced by 23.8% in Pakistan. Alpha values show the same results in Carhart4F, FF5F models with and without LMM factor. While In China the market excess return, RMRF, is reduced by 5.7% in CAPM, 2.0% in FF3F, 4.8% in FF5F while there is no change seen in Carhart 4F model. However, market factor increased in India by 1.1% while it reduced by 11.6% in Pakistan and remain same in Sri Lanka in the CAPM model. | 630-645 |
| 2. | Adjusted R-squared Values for Bangladesh  In Tables 3 through 6, the reported Adjusted R-squared values for the CAPM model (with and without the least-minus-more (LMM) frictional factor) for Bangladesh are all shown as 1.0. This appears highly implausible and warrants closer examination. Moreover, the authors should provide a detailed discussion explaining the varying explanatory power of the financial friction factor across different countries. This may include an exploration of country-specific characteristics that influence the effect of financial friction on stock returns. | Since the data of Bangladesh has a lot of autocorrelation and multicollinearity issues. Therefore, its most of the results are insignificant and implausible. We have noticed either extremely high adjusted r-square value or so negligible and zero adjusted R-square value in case of Bangladesh in most of the test and models applied. However, adjusted r-square values are corrected.  The varying explanatory power of the financial friction factor across countries can be attributed to differences in institutional and financial development as it is observed in case of China and India which are higher financially developed and are less prone to these frictions as firms have greater access to diverse funding sources (16). However, Pakistan, Bangladesh and Sri Lanka are less financially developed and have more financial frictions. Countries with more mature legal systems and higher investor protections tend to show lower financial friction effects on stock returns. Moreover, country-specific characteristics such as institutional quality, corporate governance, creditor rights, market competitiveness, and macroeconomic factors that influence these effects. For instance, better corporate governance and institutional quality tend to reduce financial transaction costs and the small-firm premium, affecting the explanatory power of financial frictions on stock returns differently by countries by their diverse macroeconomic factors. | 362-373 |
| 3. | Measurement of Financial Frictions  The approach to measuring financial frictions requires further elaboration in both the literature review and methodology sections. A more comprehensive discussion of existing theories and methodologies related to financial frictions is needed, along with a clear justification for the authors' chosen method. This will strengthen the theoretical grounding of the study and reinforce its methodological rigor. | While prior research has proxied macroeconomic frictions using variables such as oil prices (17), exchange rate risk (18), tariffs and transportation costs (19), the mortgage debt-to-income ratio (20) , the cost of debt, and price and wage stickiness (21), however, the loan-to-net asset ratio appears to be the superior indicator for capital-intensive manufacturing firms in emerging Asian economies. Consequently, the total debt-to-net assets ratio is adopted here as a proxy for macroeconomic frictions, reflecting the significance of debt as a primary capital requirement relative to a firm's net assets. This choice is further justified by the inclusion of both small and large firms in the sample, precluding the separate consideration of total debt.  Prior research has measured microeconomic frictions through indicators like risk-weighted capital requirements, bank holdings of safe securities, and risk-based capital requirements (22), alongside trade credit and collateral constraints (23, 24), and the restrictive effects of labor regulation on industry (25, 26). Bachmann and Ma (27) also investigated microeconomic frictions by examining plant-level investment spikes. In contrast, this study quantifies microeconomic frictions in manufacturing firms using a newly developed metric. This ratio is calculated by aggregating short-term trade credits, accounts payable, and financing costs from diverse sources (suppliers, merchandisers, financial institutions, and banks), and then normalizing this sum by the firm's total asset value. The interpretation of this metric is that a higher value reflects reduced financial constraints, facilitating smoother financial operations and better access to funding. Conversely, a lower value suggests substantial financial limitations and difficulties in conducting inter-firm activities. This novel measurement of microeconomic frictions offers a significant contribution to understanding the financial well-being and operational effectiveness of manufacturing companies.  Various proxies have been employed to estimate financial market frictions, including specific fiscal taxes, real transaction costs for investors, the capital gains tax rate (28-31), and the basis (the spread between credit default swap and corporate bond rates) for a significant number of firms (32). The Treasury Eurodollar spread (TED Spread), defined as the yearly difference between interbank loan rates and government bond yields, has also been utilized as an independent variable to represent the level of financial market frictions Li, Liu (33), and Matvos, Seru (34). Furthermore, Matvos, Seru (34) measured financial market frictions using the excess bond premium (EBP), the TED Spread, and the yield differential between corporate bonds with Baa and Aaa ratings. Therefore in this study same measure of TED spread used to measre financial market friction.  The presence of firm-level frictions can be ascertained by examining constraints on cash flows, impediments to business operations (35), difficulties in achieving optimal capital adjustments, elevated labor and production costs, agency costs arising from managerial and directorial behavior, and limitations imposed by an untrained and unskilled workforce (36, 37). As an illustration, Neumeyer and Perri (38) and Mendoza and Smith (39) assessed firm-level frictions by focusing on working capital financial constraints, which compel businesses to seek borrowing to finance a portion of their wage expenditures or the prepayment of intermediate inputs. Therefore, in this study total labor, production, operations and selling cost to total assets are proxied for firm level friction. | 400-444 |
| 4 | Clarity and Precision of Language  Several portions of the manuscript contain unclear expressions and minor grammatical inaccuracies. The authors should undertake a thorough review of the text to ensure all statements are articulated clearly and are free from language errors. This will improve the overall readability and professionalism of the paper. | Many language and grammatical mistakes are removed from the complete article and refine it for better clarity and precision. Unclear expressions are removed and corrected. All the authors make a thorough review of the article at least twice and then this refined version is represented to increase professionalism and readability. | In complete article necessary changes and corrections are made. |
|  |  |  |  |

**References**

1.	Ferreira MH, Haber T, Rörig C. Financial Constraints and Firm Size: Micro-Evidence and Aggregate Implications. Queen Mary University of London, School of Economics and Finance; 2023.

2.	Bali TG, Brown SJ, Tang Y. Is economic uncertainty priced in the cross-section of stock returns? Journal of financial economics. 2017;126(3):471-89.

3.	Ganai SG, Mir AH, Bhat SA, Khan JA. Impacting instruments for export competitiveness: Evidence from India and China in the global manufacturing market. Global Journal of Emerging Market Economies. 2024;16(1):130-42.

4.	Afzal R, Salahuddin T, Bashir F, Hussain A. Impact of COVID-19 on Small and Medium Enterprises in South Asian Countries. Journal of Social Sciences Review. 2023;3(1):866-82.

5.	Fama EF, French KR. A five-factor asset pricing model. Journal of financial economics. 2015;116(1):1-22.

6.	Carhart MM. On persistence in mutual fund performance. The Journal of finance. 1997;52(1):57-82.

7.	Fama EF, French KR. Common risk factors in the returns on stocks and bonds. Journal of financial economics. 1993;33(1):3-56.

8.	Wang H. Information asymmetry and agency problems in the financial market. Highlights in Business. Economics and Management. 2024;32:62-6.

9.	Bushman RM, Smith AJ. Financial accounting information and corporate governance. Journal of accounting and Economics. 2001;32(1-3):237-333.

10.	Hadlock CJ, Pierce JR. New evidence on measuring financial constraints: Moving beyond the KZ index. The review of financial studies. 2010;23(5):1909-40.

11.	Bodie Z, Kane A, Marcus AJ, Mohanty P. Investments (SIE): McGraw-Hill Education; 2014.

12.	Pástor Ľ, Stambaugh RF. Liquidity risk and expected stock returns. Journal of Political economy. 2003;111(3):642-85.

13.	Amihud Y, Hameed A, Zhang H. Global Evidence on the Premium for Market Illiquidity. Macroeconomic Review. 2022;21:95-102.

14.	Fama EF, French KR. Comparing cross-section and time-series factor models. The Review of Financial Studies. 2020;33(5):1891-926.

15.	Khan S, Shoaib A. Firm value adjustment speed through financial friction in the presence of earnings management and productivity growth: evidence from emerging economies. Humanities and Social Sciences Communications. 2024;11(1):1-17.

16.	Levine O, Warusawitharana M. Finance and productivity growth: Firm-level evidence. Journal of Monetary Economics. 2021;117:91-107.

17.	Jain K, David S. Crude oil prices and its role in economy. International Journal of Managment, IT and Engineering. 2013;3(11):491-9.

18.	Priya P, Sharma C. Monetary policy, macroeconomic uncertainty and corporate liquid asset demand: a firm-level analysis for India. International Journal of Managerial Finance. 2023.

19.	Corlay G, Dupraz S, Labonne C, Muller A, Antonin C, Daudin G. Comment: Inferring trade costs from trade booms and trade busts. International Economics. 2017;152:1-8.

20.	Huo Z, Ríos-Rull J-V. Tightening financial frictions on households, recessions, and price reallocations. Review of Economic Dynamics. 2015;18(1):118-39.

21.	Smets F, Wouters R. Shocks and frictions in US business cycles: A Bayesian DSGE approach. American economic review. 2007;97(3):586-606.

22.	Covas F, Driscoll JC. Bank liquidity and capital regulation in general equilibrium. 2014.

23.	Castro R, Ševčík P. Occupational choice, human capital, and financial constraints. CHCP Working Paper; 2016.

24.	Peia O, Romelli D. Did financial frictions stifle R&D investment in Europe during the great recession? Journal of International Money and Finance. 2022;120:102263.

25.	Botero JC, Djankov S, Porta RL, Lopez-de-Silanes F, Shleifer A. The regulation of labor. The Quarterly Journal of Economics. 2004;119(4):1339-82.

26.	Heckman JJ, Pagés C. The cost of job security regulation: Evidence from Latin American labor markets. National bureau of economic research Cambridge, Mass., USA; 2000.

27.	Bachmann R, Ma L. Lumpy investment, lumpy inventories. Journal of Money, Credit and Banking. 2016;48(5):821-55.

28.	DeGennaro RP, Robotti C. Financial market frictions. DeGennaro, Ramon P and Cesare Robotti“Financial Market Frictions” Economic Review. 2007;92:1-16.

29.	Baker M, Wurgler J. Behavioral corporate finance: An updated survey. Handbook of the Economics of Finance. 2: Elsevier; 2013. p. 357-424.

30.	Adrian T, Etula E, Muir T. Financial intermediaries and the cross‐section of asset returns. The Journal of Finance. 2014;69(6):2557-96.

31.	Kozak S, Nagel S, Santosh S. Shrinking the cross-section. Journal of Financial Economics. 2020;135(2):271-92.

32.	Levin A, Perli R, Zakrajsek E, editors. The determinants of market frictions in the corporate market. Computing in Economics and Finance; 2005.

33.	Li S, Liu C, Zheng M, Chang C-P, Fu Q. Financial market friction and corporate restructuring activities in China. Emerging Markets Finance and Trade. 2021;57(14):4089-104.

34.	Matvos G, Seru A, Silva RC. Financial market frictions and diversification. Journal of Financial Economics. 2018;127(1):21-50.

35.	Chan JM. Financial frictions and trade intermediation: Theory and evidence. European Economic Review. 2019;119:567-93.

36.	Giroud X, Mueller HM. Firm leverage, consumer demand, and employment losses during the great recession. The Quarterly Journal of Economics. 2017;132(1):271-316.

37.	Gopinath G, Kalemli-Özcan Ş, Karabarbounis L, Villegas-Sanchez C. Capital allocation and productivity in South Europe. The Quarterly Journal of Economics. 2017;132(4):1915-67.

38.	Neumeyer PA, Perri F. Business cycles in emerging economies: the role of interest rates. Journal of monetary Economics. 2005;52(2):345-80.

39.	Mendoza E, Smith KA. Financial Frictions, Sudden Stops, and the External Capital Structure of Emerging Markets. 2008.
